# Research Progress on the NSP10 Protein of Porcine Reproductive and Respiratory Syndrome Virus

**DOI:** 10.3390/microorganisms12030553

**Published:** 2024-03-11

**Authors:** Gan Li, Yajie Zheng, Qin Luo, Yaohua Liang, Hang Zhang, Huiyang Sha, Ruining Wang, Weili Kong, Mengmeng Zhao

**Affiliations:** 1 School of Life Science and Engineering, Foshan University, Foshan 528225, China; ligan1227@163.com (G.L.); zhengyajie2022@163.com (Y.Z.); luoqin121104@163.com (Q.L.); hangzh2022@163.com (H.Z.); huiyangsha2022@163.com (H.S.); 2 Department of Agricultural and Biosystems Engineering, South Dakota State University, Brookings, SD 57007, USA; yaohua.liang@sdstate.edu; 3 College of Veterinary Medicine, Henan University of Animal Husbandry and Economy, Zhengzhou 450046, China; 80882@hnuahe.edu.cn; 4 Gladstone Institutes of Virology and Immunology, University of California, San Francisco, CA 94158, USA; weili.kong@gladstone.ucsf.edu

**Keywords:** PRRSV, NSP10, helicase, genetic evolution, apoptosis, viral virulence

## Abstract

Porcine reproductive and respiratory syndrome (PRRS) is a highly contagious and pathogenic infectious disease caused by the porcine reproductive and respiratory syndrome virus (PRRSV). It manifests as reproductive disorders in sows and respiratory disorders in piglets. PRRSV infects swine herds with symptoms such as abortions, stillbirths, and mummified fetuses in gestating sows. Piglets mainly experience abdominal respiration and respiratory symptoms. To date, the prevention of PRRS relies primarily on vaccination and the implementation of various preventive and control measures. Swine deaths caused by PRRS have resulted in significant economic losses to the pig industry worldwide. Non-structural protein 10 (NSP10) has helicase and adenosine triphosphatase (ATPase) activities that unwind DNA and RNA and play important roles in viral replication and transcription. Hence, it can be potentially used to develop novel reagents for the detection of PPRSV. This article reviews genetic variations, interaction with viral and host proteins, effects on PRRSV replication, immunomodulation, apoptosis, and viral virulence of NSP10, with the aim of providing a theoretical basis for the prevention and control of PRRS and drug development in the future.

## 1. Introduction

Porcine reproductive and respiratory syndrome (PRRS), commonly known as “Blue Ear Disease”, is a highly contagious and pathogenic disease caused by the porcine reproductive and respiratory syndrome virus (PRRSV), which is mainly responsible for respiratory diseases in pigs of all ages and reproductive disorders in sows. Consequently, this disease poses a threat to the global pig industry.

PRRS first occurred in the United States in 1987 [1], and adversely affected countries such as the Netherlands and Japan in the 1990s [2,3], after which this disease spread rapidly across Europe and Asia. Currently, it is endemic worldwide and causes significant economic losses to the swine industry. PRRSV is categorized into two species, *Betaarterivirus suid 1* (PRRSV-1) and *Betaarterivirus suid 2* (PRRSV-2), which share approximately 60% nucleotide identity [4]. The PRRSV genome exhibits a high frequency of mutations and recombination events [5], and has produced several novel strains since its discovery, such as the highly pathogenic PRRSV (HP-PRRSV), NADC30, and NADC34 strains, which have been widely disseminated in swine populations in recent years [6,7,8]. There are no specific drugs available to treat PRRS, rendering it a prominent infectious disease within the swine industry.

PRRSV primarily infects porcine alveolar macrophages (PAMs) [9]. It is a positive-sense single-stranded RNA virus with a lipid bilayer envelope and well-defined gene sequence, with a genome length of approximately 15.4 kb and 11 open reading frames (ORFs); ORF1a and ORF1b account for approximately 80% of the genome and encode the polyproteins pp1a and pp1ab, respectively, which are hydrolyzed by proteases into 16 different non-structural proteins (NSPs) [10,11,12,13,14]. Under the action of NSP4, which possesses 3C-like serine protease activity, pp1ab is hydrolyzed to form four highly conserved NSPs: NSP9, NSP10, NSP11, and NSP12 [15]. NSP10 has helicase and adenosine triphosphatase (ATPase) activities, which can unwind double-stranded RNAs (dsRNAs) and drive viral genome replication, subgenomic mRNA transcription, viral particle generation, and subgenomic RNA (sgRNA) synthesis [16,17,18,19]. Hence, it is a factor that plays an important role in the regulation of PRRSV synthesis.

**Figure 1:**
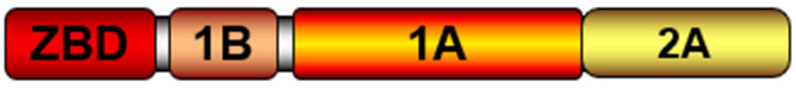
Schematic structure of PRRSV NSP10. The structure is composed of an N-terminus containing a zinc-binding domain (ZBD), a 1B domain in the middle, and C-terminal helicase core structural domains 1A and 2A.

NSP10 consists of an N-terminal zinc-binding domain (ZBD), 1B domain, and C-terminal helicase core structural domains 1A and 2A [20] (Figure 1). These structural domains are extremely important for the unwinding activity of NSP10. Specifically, the N-terminal ZBD can control the activity of helicase and ATPase, and C^25^ and H^32^ amino acids on the ZBD are key sites that affect protein binding and unwinding activities, but do not affect ATPase activity [21]. Although ZBD residues are not catalytically active, they can help NSP10 fold and bind better to substrate RNA molecules [22]. Deletion of 1B and C-terminal structural domains significantly reduces the unwinding activity of NSP10 [20]; both of these structural domains affect the synthesis of PRRSV. NSP10 controls the synthesis of viral RNA and is a molecule that can be potentially employed to detect PRRSV. Thus, analysis of its structure and function can further elucidate the functional mechanism of helicases.

This article comprehensively reviews NSP10, encompassing an analysis of its genetic variation, interaction with viral and host proteins, effects on viral replication, regulation of the host immune response, induction of apoptosis, and impact on virulence, thereby, laying a theoretical foundation aimed at prevention and control, vaccine design, and the development of drugs against PRRS.

## 2. Analysis of Genetic Variation of NSP10

The *NSP10* gene is 1320 bp in length and consists of seven conserved motifs; its helicase activity is heat-resistant, and its nucleoside triphosphate hydrolase (NTPase) activity is sensitive to changes in pH, making it one of the most conserved proteins encoded by ORF1b [17,23]. Wang [24] sequenced the whole genome of the PRRSV A2MC2 strain and identified six amino acid changes distinct from those found in the VR-2332 and MLV strains. These alterations were located in NSP8, NSP10, NSP12, and GP3, with two amino acid changes occurring in the NSP10 protein. Zhou et al. [23,25] demonstrated the presence of nucleotide substitutions, namely A → G or T → C transitions, in the *NSP10* gene. They found that the nucleotide identity of the *NSP10* gene between the PRRSV JL strain and highly pathogenic HN2007, TJ, and GS2008 strains was over 98.3%, while that between the PRRSV JL strain and strains MLV, BJ-4, and GS2004 was 91.7–94%. Despite variations in nucleotide identity among these strains, amino acid identity was over 97.4%. Xu et al. [26] compared the nucleotide and amino acid sequences of the NADC34-like strains with those of the NADC30 and ISU30 strains. The results revealed that the nucleotide identity of the *NSP10* gene of the NADC34-like NADC30 strains ranged from 90.6% to 92.2%, and that with the ISU30 strains ranged from 90.8% to 92.4%, whereas the amino acid identity among all the strains was above 97.7%. Chen et al. [27] compared the identity of the HBap4-2018 strain with JXA1, HuN4, VR2332, CH-1a, NADC30, and CHsx1401 strains. They found that the nucleotide identity of the *NSP10* gene between the HBap4-2018 strain and these virulent strains ranged from 84.7% to 96.7%, and the amino acid identity ranged from 94.8% to 98.2%. Taken together, these studies suggest a high degree of conservation within the *NSP10* gene.

NSP10 is involved in recombination events in virulent strains. Li [28] discovered that recombination break regions related to potential recombination events observed in the SDYT-p13-2013 and SDGM-7-3-2016 strains involved the *NSP10* gene. Zhou et al. [29] reported that a recombination event in the NADC30-like SCcd17 strain involved a portion of the *NSP10* gene. Sun et al. [30] discovered the TZJ2134 strain in non-vaccinated aborted sows from a small backyard farm in Shandong Province, China. Through BLAST analysis, they found that the TZJ2134 strain was created through a recombination event between the Amervac and DV vaccine strains and that this recombination breakpoint was located in NSP10. Leng et al. [31] analyzed the full nucleotide sequence of the TJ vaccine strain and found that the 31 amino acid mutations that occurred in this strain were distributed in the NSP1β, NSP2-NSP5, NSP7, NSP9, NSP10, GP4, and GP5 regions. Thus, it is clear that recombination or mutation of the *NSP10* gene may lead to the formation of novel virulent strains. In addition, Chao et al. [32] identified seven novel subgenomic defective interfering (DI) RNAs in PRRSV that had deletions ranging from 8513 to 9176 nucleotides between NSP1/NSP2 and NSP10. The discovery of DI RNAs provides new insights into the proliferation of PRRSV.

## 3. NSP10 Interacts with PRRSV Proteins

Song and Nan et al. [33,34] mapped the interaction network among PRRSV NSPs, indicating that the centers of these interactions are located on ORF1b-encoded NSP9, NSP10, and NSP12. These proteins participate in the assembly of replication and transcription complexes (RTCs), which bind to the cell membrane with the support of a scaffold formed by the joint binding of transmembrane proteins NSP2, NSP3, and NSP5, thereby regulating viral replication and transcription. By using a yeast two-hybrid (Y2H) assay, it was discovered that NSP10 interacts with NSP1α and co-localizes in the cytoplasm. Moreover, co-transfection of cells revealed that NSP10, containing a helicase domain (128–441 aa), interacts with NSP2, exhibiting delocalization and co-localization from the nucleus to the cytoplasm. It was also found that the 1B domain affected the helicase domain, significantly improving the delocalization efficiency. Similar results were obtained for interactions between NSP10 and NSP3 as well as between NSP10 and NSP5. NSP2, NSP3, and NSP5 could relocate only the C-terminal structural domain of NSP10, whereas NSP12 could relocate both the C- and N-terminal structural domains of NSP10. Deletion of 124 to 133 aa in the linkage region of structural domains 1A and 1B of NSP10 enabled NSP12 to relocate the C- and N-terminal structural domains of NSP10. The occurrence of a single amino acid mutation (E^131^A, I^132^A) in the structural domain of NSP10 can activate interaction between NSP10 and NSP12, consequently impeding the accumulation of small guide RNA (sgRNA) [35]. Furthermore, NSP10 can also interact with NSP9. The binding of NSP10 and NSP9 to membrane-associated NSPs may be involved in the regulatory mechanisms of other viral proteins during infection [33]. In addition, NSP10 interacts with the nucleocapsid (N) protein of PRRSV, which induces the expression of a dendritic cell marker, CD83, and co-regulates viral proliferation in the host [36].

**Figure 2:**
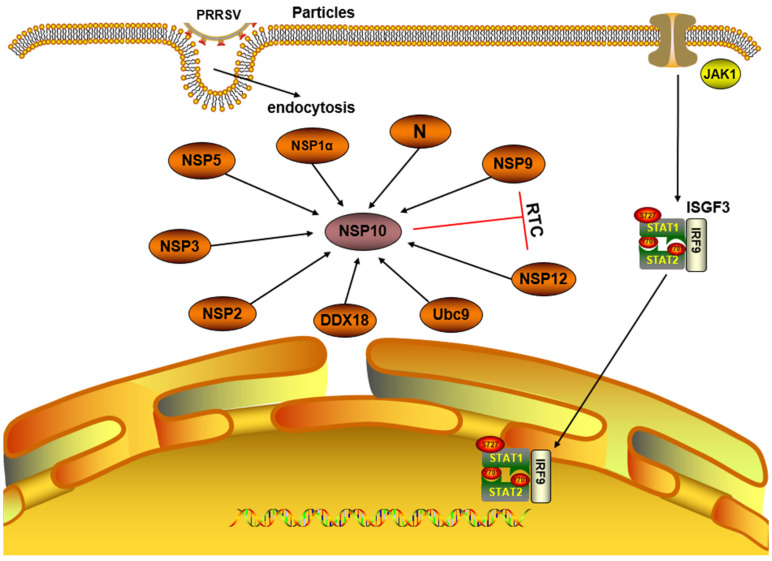
Schematic representation of PRRSV NSP10 interacting with viral and host proteins. Host cells activate the JAK/STAT pathway upon infection with PRRSV. NSP10, NSP9, and NSP12 are involved in the formation of key parts of the transcription complexes (RTCs).

In summary, NSP10 of PRRSV interacts with NSP1α, NSP2, NSP3, NSP5, NSP9, NSP12, and N proteins, all of which are jointly involved in the transcriptional replication of the viral genome. Specific interactions of NSP10 with viral and host proteins are shown in Figure 2, which may provide novel concepts for research and the development of new anti-viral drugs.

## 4. NSP10 Interacts with Host Proteins

PRRSV interacts with host proteins to create a more suitable environment for replication [37]. Among the currently available studies, only the DEAD-box RNA helicase 18 (DDX18) and SUMO E2 conjugating enzyme Ubc9 are known to interact with NSP10. DDX18, which belongs to the RNA helicase superfamily 2, possesses nine conserved motifs that govern ATPase and helicase activities [38,39,40]. It plays an important role in the maturation and translation of viral RNAs. Jin et al. [41] employed the co-immunoprecipitation (Co-IP) technique to demonstrate that the C-terminal domain of DDX18 could interact with the N-terminal ZBD and C-terminus domains of NSP10. NSP10 induces the translocation of DDX18 from the nucleus to the cytoplasm, which re-mediates the process of RNA replication. Overexpression of DDX18 positively regulates PRRSV proliferation; conversely, interference with the gene expression of the DDX18 protein significantly inhibits RNA replication. Wang et al. [42] demonstrated that NSP10 interacts and colocalizes with Ubc9 in the cytoplasm. Overexpression of Ubc9 during the early stages of PRRSV infection inhibits viral genome replication, whereas RNA synthesis is promoted by knocking down Ubc9 using small interfering RNA (siRNA). Interaction between NSP10 and host proteins influences the proliferation of PRRSV. At the same time, Dong [43] reported that although NSP10 down-regulates the expression of interferon regulatory factor 1 (IRF-1), it does not interact with IRF-1. A study found that bone marrow stromal cell antigen 2 (BST2) inhibits the expression of NSP10 [44]; however, in-depth studies are required to explore whether it interacts with NSP10. Few studies have investigated the interaction between NSP10 and host proteins, and its mechanisms of action and applications need to be further investigated.

## 5. Impact of NSP10 on Viral Replication

NSP10 directs viral genome replication and the synthesis of subgenomes, which occur within the double-membrane structure of the viral replication complex [45]. NSP10, the helicase of PRRSV, belongs to the SFB1 superfamily and is responsible for encoding synthetic RNAs that use the energy generated by adenosine triphosphate (ATP) hydrolysis to unravel the viral RNA double strand in vivo, with unwinding occurring from the 5’-end to the 3’-end [17,46]. NSP9 is an RNA-dependent RNA polymerase (RdRP) encoded by ORF1b, an essential protein for viral genome replication and the synthesis of subgenomes [47]. It has been shown that NSP10 alone does not activate the replicative subsystem of PRRSV and that co-expression with NSP9 does not activate this replicative system to synthesize negative-stranded RNA [48]. This suggests that PRRSV replication is co-regulated by multiple proteins and that individual proteins cannot regulate viral proliferation. In addition, Li et al. [49] discovered that the combined action of NSP10 and NSP9 affected the replication efficiency of HP-PRRSV strains. Thus, NSP10 and NSP9 together constitute key enzymes for PRRSV replication and transcription.

NSP10 is capable of activating the Wnt/β-catenin signaling pathway, and this pathway inhibits PRRSV replication [50]. The N-terminal ZBD of the *NSP10* gene encodes 13 conserved cysteine (Cys) and histidine (His) residues, and mutations at the Cys^25^ and His^32^ sites eliminate the unwinding activity and affect the regulation of viral replication by NSP10 [16,21]. The substitution of a single base or mutation of the entire structure in ZBD leads to the functional impairment of NSP10, and the inactivation of the function of NSP10 cannot be restored by wild-type ZBD complementation in trans, suggesting that ZBD exerts a cis-regulatory role in NSP10 [22]. The amino acid sequence of NSP10 contains several sites associated with unwinding activity and ATP hydrolyzing activity. Mutations at these sites result in a decreased function of NSP10 and reduced efficiency of viral replication and synthesis. Zhang et al. [21] demonstrated that Lys^155^ (K^155^) is a critical amino acid residue for the ATPase activity of NSP10, while Ala^227^ (A^277^) is a key amino acid residue for helicase activity. Mutations at both of these sites attenuated ATP hydrolyzing and uncoupling activities. Liu et al. [51] discovered that a mutation (His^313^ to Tyr^313^) occurs in NSP10 during the passaging of PRRSV. Whether this mutation affects the unwinding activity, thereby affecting replication and synthesis of the virus, needs to be explored further.

Mutations or substitutions in certain segments of NSP10 can increase the replication rate of the viral genome. Jiang [52] constructed a recombinant strain, rHuN4-F112-NSP10, by replacing the NSP10 of the HP-PRRSV HuN4 strain with a corresponding fragment in the attenuated vaccine strain HuN4-F112 using reverse genetics. Two mutations were found to occur in the N-terminal ZBD of NSP10, which increased the replication rate of the virus. NSP10 influences RNA replication by affecting certain viral structures. Fang et al. [53] demonstrated that NSP10 could unwind the G-quadruplex (G4) structure of the negative-stranded RNA of PRRSV and promote viral synthesis. Chen [54] designed three siRNAs against the *NSP10* gene and screened three target sites that could inhibit viral replication. Zhang et al. [55] examined cells infected with the HP-PRRSV JXwn06 strain and identified a truncated form of NSP10, designated as NSP10a, which was formed because of the deletion of approximately 70 aa at the N-terminus of NSP10. This study confirmed that the production of NSP10a was not associated with the proliferation of PRRSV. The *NSP10* gene, which plays the role of a helicase, appears only during the process of viral transcription and replication. The presence of NSP10 implies that pigs are infected with PRRSV, making it a crucial molecule that can be employed for the detection of PRRSV and for antiviral drug research. In the future, novel strategies can be developed to detect PRRSV and treat PRRS by studying NSP10.

## 6. NSP10 Regulates the Host Immune Response

PRRSV mainly invades PAMs, inducing the host to secrete a large number of inflammatory factors, such as the NOD-like receptor protein 3 (NLRP3) inflammasome, which suppresses the innate immune responses of the host and leads to persistent infections [56,57,58]. PRRSV also infects the animals concurrently with other viruses or bacteria, thus posing a great challenge to the treatment of PRRS. Song et al. [59] observed that NSP10 exerts an inhibitory effect on NLRP3, thereby inhibiting the inflammatory response. NSPs are involved in anti-host innate immunity, including humoral and cellular immunity. NSP10 primarily participates in the cellular immune responses generated by T lymphocytes in animals. CD83 is a dendritic cell (DC) marker that belongs to the immunoglobulin (Ig) superfamily and exhibits two isoforms: membrane-bound CD83 (mCD83) and soluble CD83 (sCD83); sCD83 inhibits T cell proliferation and interferes with the maturation of DCs and the cytoskeleton [60,61]. Chen et al. [36] found that after PRRSV infection of swine herds, NSP10 induced the expression of sCD83 in porcine monocyte-derived dendritic cells (MoDCs) through the nuclear factor-kappa B (NF-κB) and specificity protein 1 (Sp1) signaling pathways, which interfered with T cell proliferation and inhibited the production of interleukin 2 (IL-2) and interferon-γ (IFN-γ) by T cells. This study also demonstrated that structural domains P192-5 and G214-3 of the NSP10 protein play important roles in regulating CD83 signaling, which significantly enhances the activity of the CD83 promoter, thereby suppressing the immune response of host cells and promoting PRRSV proliferation.

Zhou et al. [62] cloned the *NSP10* gene of the virulent HP-PRRSV JL strain and constructed a recombinant expression vector, pET-32a/NSP10. They evaluated the expression and immunoreactivity of the NSP10 protein using sodium dodecyl sulfate-polyacrylamide gel electrophoresis (SDS-PAGE) and Western blotting, and demonstrated that the NSP10 protein was successfully expressed and had favorable immunogenicity. Interleukin 10 (IL-10) is an immune-regulatory-related cytokine that plays an important role in the immunosuppressive mechanism of PRRSV infections [63,64]. NSP10, as a factor that affects viral proliferation, influences viral immunosuppression by decreasing the expression of IL-10. Jiang et al. [65] constructed a chimeric virus, rHuN4-F5-ORF1b, and found the expression of IL-10 decreased after cells were infected with the virus. They speculated that this effect was mediated by viral replication and the transcription-associated factors NSP9 and NSP10. Parida et al. [66] found that the NSP10 sequence contains two highly conserved T cell epitopes and has the ability to induce proliferation and an IFN-γ response in peripheral blood monocytes, which is important to elicit an immune response in the body to produce a cross-protective effect against different PRRSV variant strains. After PRRSV infection in swine, there is a significant increase in the expression of IFN-γ, indicating an elevation in cytotoxic T lymphocytes (CTLs) in the animals as a means of resisting invasion by the virus. Dong [43] co-transfected HEK293FT cells with IRF-1 and NSP10 to validate that NSP10 could degrade IRF-1 through proteasome and lysosomal pathways, and regulate the IFN-γ-mediated signaling pathway, thus promoting the proliferation of PRRSV. Furthermore, NSP10 expression can be effectively inhibited by matrine and glycyrrhizic acid, which mobilize the immune system to resist PRRSV infection [67] and have the potential to be used as an anti-PRRSV formulation.

Investigating the mechanisms underlying the immunosuppressive effects of NSP10 could provide scientific evidence for the development of new antiviral drugs, thus paving the way to treat PRRS in the future.

## 7. Induction of Apoptosis by NSP10

PRRSV primarily infects PAMs and induces apoptosis [68,69]. It has been shown that PRRSV infection of Marc-145 cells, which are derived from African green monkey kidney cells, can regulate apoptosis at different periods of infection [70]. Yuan et al. [71] confirmed that NSP10 serves as a pro-apoptotic factor, activating caspase-8 and caspase-9 in PRRSV-infected Marc-145 cells through exogenous death receptors and endogenous mitochondria-dependent pathways, thereby regulating apoptosis in host cells. NSP10-induced apoptosis is also associated with the B-cell lymphoma-2 (Bcl-2) family. The Bcl-2 family is involved in PRRSV-mediated apoptosis. Members of this family include pro-apoptotic and anti-apoptotic factors, with the main pro-apoptotic factors being BH3-interacting domain death agonist (Bid), Bcl-2-interacting mediator of cell death (Bim), Bcl2-associated X protein (Bax), and B-cell lymphoma-extra-large (Bcl-xL) [72,73]. The Bim subtype, Bims, is present in NSP3, NSP4, and NSP10 and antagonizes, the anti-apoptotic function of Bcl-xL [71,74]. Death receptor-mediated activation of caspase-8 cleaves Bid produces a pro-apoptotic active form of truncated Bid (tBid) that is produced only in the presence of NSP10, and its expression is significantly increased by NSP10, whereas caspase-8 promotes apoptosis by connecting endogenous and exogenous apoptotic pathways through Bid [71,72]. A study suggested replacing the NSP10 fragments of different pathogenic strains and analyzing the differences in host cell apoptosis. The results demonstrated that NSP10 predominantly determines differences in apoptosis among different viral strains [75]. These findings provide a scientific basis related to apoptosis for the understanding of pathogenesis of PRRSV.

## 8. Effect of NSP10 on the Pathogenicity of PRRSV

NSP10 affects the pathogenicity of PRRSV only in conjunction with other NSPs. It is possible that NSP10 alone may influence the early growth of virus-infected host cells without affecting virulence [52]. Know et al. [76] constructed chimeric viruses of FL-12 and PP vaccine strains, and demonstrated that NSP3-8 and ORF5 were the major pathogenic factors of PRRSV, with NSP1-3, NSP10-12, and ORF2 as minor pathogenic factors.

In the prevention and control of PRRS, attenuated vaccines used in vaccination can sometimes regain their pathogenicity. This phenomenon typically arises because of revertant genomic mutations, compensatory mutations, recombination, or quasi-species diversity changes in PRRSV that alter the normal genome sequence [77]. Liu et al. [51] evaluated the pathogenicity rebound phenomenon of two candidate strains of HP-PRRSV-attenuated vaccines that appeared after the passaging of the strains in vivo. The revertant mutations in NSP1β, GP5a, and NSP10 were identified to be associated with HP-PRRSV pathogenicity. This study also hypothesized that the revertant mutations in NSP1β and NSP10 were associated with pathogenicity potentiation. Henriette et al. [78] analyzed three wild isolates derived after the application of a weakly virulent vaccine to a Danish field and identified two revertant mutations occurring in NSP1β (Phe-Ser) and NSP10 (His-Tyr). They speculated that these mutations could potentially be associated with the restoration of attenuated vaccine pathogenicity. These genomic revertant mutations accumulated during in vivo transmission, ultimately resulting in the reversal of the pathogenicity of the attenuated vaccine strain.

Mutations at amino acid sites of NSP10 are associated with viral pathogenicity. Allende et al. [79] found that the Y^952^ site in NSP10 is associated with pathogenicity. Mutations at this site may reduce the efficiency of RNA unwinding and disrupt viral replication, consequently leading to attenuation of pathogenicity. Leng et al. [31] investigated attenuation of pathogenicity in the TJ strain and, revealed that out of 31 amino acid mutations occurring in this strain, 4 mutations (V^296^M, S^297^A, K^386^R, and V^436^I) were located in NSP10. It was hypothesized that the amino acid change in NSP10 is one of the potential effects of the attenuation of PRRSV pathogenicity; however, evidence to substantiate this attenuation requires further study. When analyzing the genome of the low-pathogenicity strain GDQJ, Li et al. [80] identified multiple genomic mutations in the 5’UTR, NSP1β, NSP2, NSP3, NSP5, NSP7, NSP9, NSP10, GP5, and N regions, among which there was a substitution of the amino acid V^299^ → A^299^ in NSP10. This provides evidence that amino acid mutations in NSP10 affect the virulence level of PRRSV. Wei [81] passaged the low-pathogenicity HNLY1707 strain for 100 consecutive passages, performed a genome sequence analysis, and found 31 amino acid mutations that could be stably inherited, including one mutation (K^408^E) in the amino acid sequence of NSP10. Chen et al. [82] transmitted the HP-PRRSV XH-GD strain for 122 consecutive passages in Marc-145 cells. The pathogenicity of the strain was attenuated, and 35 amino acid mutations were observed, including the amino acid mutation H^800^Y in NSP10. Wei et al. [83] passaged the PRRSV-HBR strain for 125 consecutive generations, resulting in the attenuation of virulence and multiple mutations in the amino acid sequence, with three amino acid mutations occurring in NSP10 (D^970^G, A^844^V, and R^1051^H). Yu et al. [84] performed a genomic analysis of the HP-PRRSV JXA1 strain after successive passages of the 5th and 100th–170th generations. The results revealed that, except for a revertant mutation at position 1136 in the amino acid sequence of NSP10, the high pathogenicity of the strain was attenuated, and 35 amino acid mutations were observed, including the amino acid mutation H^800^Y in NSP10. Virus (JXA1, 5th generation) mutations in the NSPs encoded by ORF1b that were present in the attenuated viruses (JXA1, 80th to 170th generation) were conserved. From the above studies, it can be speculated that NSP10 both enhances and attenuates pathogenicity. It has been hypothesized that the ability of NSP10 to enhance pathogenicity is due to revertant mutations in the genome. The emergence and accumulation of revertant mutations can increase or revert the virulence of the attenuated vaccine. NSP10 reduces the pathogenicity of the viral strain through the occurrence of amino acid mutations in the genome that are not revertant. Therefore, NSP10 does not enhance viral pathogenicity, and the pathogenicity gradually declines as the number of passages of the virus increases.

Chen [54] analyzed the amino acid identity of NSP10 across strains JXwn06, JXA1, HuN4, and WUH1 and screened two amino acid sites at positions 408 and 410 that were associated with pathogenicity. By analyzing the PRRSV-1 GZ11-G1 strain, Wang [85] revealed that amino acids at positions 261, 304, and 401 of NSP10 correlated with the pathogenicity of this strain. Li et al. [49] showed that replacement of the genes of the highly pathogenic strain RvJXwn *NSP9 + NSP10* with a low pathogenic strain led to a decrease in the proliferative capacity of the virus and the loss of lethal virulence. Thus, NSP9 and NSP10 are virulence factors of HP-PRRSV, which together lead to its high pathogenicity.

In summary, multiple amino acid sites in NSP10 are related to PRRSV pathogenicity. NSP10 alone does not affect pathogenicity, while NSP10 in conjunction with NSP9 collectively influences the pathogenicity of the virus. As a potential antiviral target of PRRSV, analyzing the mechanism underlying the effects of NSP10 on the pathogenicity of PRRSV can help control PRRS.

## 9. Application of NSP10 for PRRSV Detection

NSP10 appears only during viral replication and synthesis, and its presence implies PRRSV infection. The amino acid sequence of NSP10 was analyzed using the Jameson-Wolf method, which revealed the presence of multiple antigenic epitopes on NSP10, including two T cell epitopes and one B-cell antigenic epitope, suggesting that it possesses good immunogenicity and can be used as a target for the development of assay reagents [23,66,86]. The data also signify that NSP10 is a candidate protein that can be potentially employed for the detection of PRRSV.

Zhang et al. [86] prepared an NSP10-specific monoclonal antibody, 4D9, and employed this antibody for the identification of antigenic epitopes using confocal microscopy and Western blotting. The results indicated that the sequence ^286^AIQPDYRDKL^295^ of the B-cell antigenic epitope was recognized by 4D9. They further confirmed that the monoclonal antibody 4D9 could only recognize PRRSV-2 but not PRRSV-1. Hence, this antibody helps establish a test to distinguish between PRRSV-1 and PRRSV-2 strains. Using NSP10 to design specialized primers, two different strains can be detected simultaneously. Zhang et al. [87] designed primers for a part of the NSP10 region of PRRSV LV and VR2332 strains and established a dual-PCR method for the simultaneous detection of the two types of PRRSV strains, which reduced the detection workload. Feng [88] established an indirect ELISA using a purified NSP10 recombinant protein as an antigen. This assay has an excellent sensitivity, stability, and compliance rate compared to the IDEXX Kit and reduces non-specific reactions caused by exogenous microbial contamination. Further research on the structure of NSP10 can help develop a more rapid, sensitive, and stable assay for the timely detection of PRRSV infections and reduce economic losses.

## 10. Summary and Perspectives

NSP10 is a structurally conserved helicase that is required for the synthesis of PRRSVand has ATP hydrolysis and RNA unwinding activities. It co-regulates viral replication and transcription by interacting with viral and host proteins. As a pro-apoptotic factor, NSP10 regulates host cell apoptosis through death receptor- and mitochondria-dependent pathways and also determines differences in apoptosis among different strains, which can provide a more scientific basis for understanding the pathogenesis of PRRSV. NSP10 inhibits the immune response, and several key sites in its amino acid sequence are related to viral virulence. Mutations in these sites result in differences in the virulence of various strains. In addition, multiple antigenic sites exist on the NSP10 protein, which can be used as potential regions for the detection of PRRSV. Recombinant NSP10 has high sensitivity, stability, and specificity; these features are important for developing more economical NSP10-targeted assays.

NSP10 plays an important role in the proliferation of PRRSV, and inhibiting its function is instrumental in controlling the replication of PRRSV. In-depth studies related to the protein structure and function of NSP10 can further elucidate the functional mechanisms of helicase. In the future, NSP10 can be used as a target for the development of commercial vaccines and new antiviral drugs that may interfere with the expression of this protein. These vaccines and drugs may inhibit viral replication and transcription at the molecular level, attenuate strain virulence, and reduce immunosuppressive and apoptosis-promoting effects on the host, thus eradicating PRRS and reducing economic losses.

## Data Availability

All datasets are available in the main manuscript. The dataset supporting the conclusions of this article is included within the article.
